# The Association Between Environmental Perchlorate, Nitrate, and Thiocyanate Exposure and Oral Pain in NHANES

**DOI:** 10.3389/fpubh.2022.829466

**Published:** 2022-03-10

**Authors:** Jintao Yu, Jiawen Guo, Hengguo Zhang, Xu Cheng

**Affiliations:** ^1^Key Laboratory of Oral Diseases Research of Anhui Province, College and Hospital of Stomatology, Anhui Medical University, Hefei, China; ^2^Department of Prosthodontics, Guanghua School of Stomatology, Hospital of Stomatology, Guangdong Provincial Key Laboratory of Stomatology, Sun Yat-sen University, Guangzhou, China

**Keywords:** perchlorate, nitrate, thiocyanate, oral pain, productivity loss

## Abstract

**Aim:**

To examine the human exposure to perchlorate, nitrate, and thiocyanate, and their associations with oral pain (OP) in the general population from the U.S.

**Methods:**

A total of 13,554 participants were enrolled in the National Health and Nutrition Examination Survey. The urinary perchlorate, nitrate, and thiocyanate were measured using ion chromatography coupled with an electrospray tandem mass spectrometry. The multivariable linear and logistic regressions were performed to explore the associations of the urinary perchlorate, nitrate, and thiocyanate, with the prevalence of oral pain. Restricted cubic splines were used to explore the non-linearity.

**Results:**

There are 3,129 OP cases. There was a higher urinary level of perchlorate, nitrate, and thiocyanate in OP. We found that urinary thiocyanate was positively associated with OP (odds ratio [OR] = 1.06; [1, 1.13]; *p* = 0.049). Restricted cubic spines revealed that urinary thiocyanate was in a U-shape association with OP.

**Conclusions:**

Urinary thiocyanate was in a U-shape association with OP, suggesting that we should keep the exposure of thiocyanate under a reasonable range.

## Introduction

Chronic oral diseases consist of dental caries and periodontal disease, which affect more than 3,500,000,000 individuals (1). Oral pain (OP) is a common clinical symptom of oral disease, which influenced oral functions and overall health (2). It eventually led to higher treatment costs for the government, health insurance companies, and individuals (2). However, little is known about the factors that are associated with OP in adults.

Perchlorate, thiocyanate, and nitrate are thyroid-disrupting chemicals (3). Milk and certain plants may be the main intake source of perchlorate for humans (4). Nitrate is commonly detected in contaminated water and processed meats (5). Thiocyanate is a metabolite of cyanide in tobacco, affecting human saliva and oral mucosa (6). Studies have shown that dairy milk, meats, and groundwater could be the main source of these chemicals (7, 8). The urinary levels of these three chemicals are widely used as biomarkers to assess their exposure status (9). Accumulating evidence has suggested that perchlorate, nitrate, and thiocyanate could affect thyroid function (9) and liver function (10), and can be related to obesity (11), and cancers (12). However, the association between perchlorate, nitrate, and thiocyanate exposure and the OP in the general population is not yet clear.

In this study, we explored the association between the urinary levels of perchlorate, nitrate, and thiocyanate and the OP among the general population.

## Methods

### Study Population

**Figure 1:**
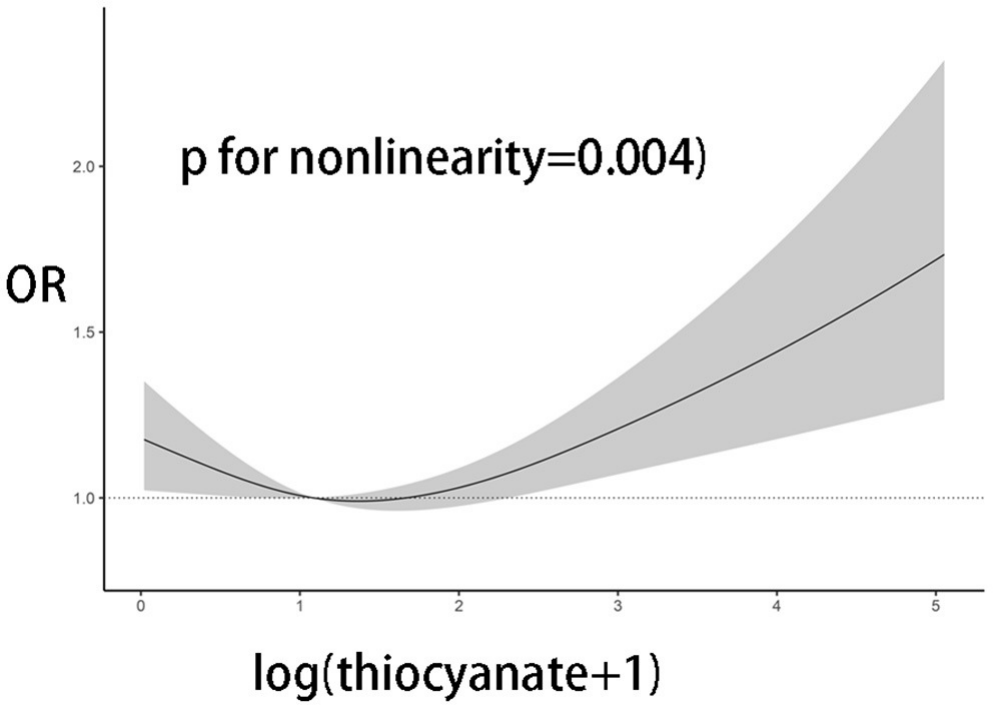
The flow chart of participant selection.

The study used data from the National Health and Nutrition Examination Survey (NHANES) cycle of 2005–2014. A total of 20,436 participants with complete records of the three chemicals were available. After excluding the participants with missing data on OP (*n* = 6,880) and the urinary creatinine (*n* = 2), 13,554 participants were enrolled in the study (Figure 1). The study was approved by the review board of the National Center for Health Statistics.

### Exposure Variable and Outcomes

Urine specimens were collected by the professional staff. Urinary levels of perchlorate, nitrate, and thiocyanate were measured by ion chromatography coupled with electrospray tandem mass spectrometry (https://wwwn.cdc.gov/Nchs/Nhanes/2013-2014/PERNT_H.htm).

Oral pain was diagnosed based on the responses to the question. The individuals who reported feeling the pain in the mouth, with frequencies, such as “very often,” “fairly often,” and “occasionally” last year, were grouped into the OP group, while those who answered “hardly ever” or “never” formed the non-OP group.

### Covariates' Collection

The baseline information was ascertained from the questionnaires, including gender, age, ethnicity/race, education, and poverty-income ratio (PIR). The body mass index (BMI) was determined by body weight and height. Urinary creatinine was also considered due to its role of being a dilution-dependent sample variation in urine concentrations (13). Urinary creatinine was determined by a Jaffe rate reaction for NHANES 2005–2006 and by an enzymatic method for NHANES 2007–2014 (14). Hypertension was defined as: (1) previous diagnosis of hypertension; or (2) blood pressure ≥ 140/90 mmHg; or (3) receiving anti-hypertensive drugs. Diabetes was defined as: (1) previous diagnosis of diabetes; (2) fasting plasma glucose ≥ 7 mmol/L, HbA1c ≥ 6.5%; or (3) taking anti-hyperglycemic drugs. Multiple imputations using a predictive mean matching (PMM) were performed for covariates with missing values.

### Statistical Analysis

The variables were presented as numbers (percentage), and the mean (standard deviation) or the median (lower quartile, upper quartile) as appropriate. Differences between groups were explored using Students' *t*-test or the Mann–Whitney *U-*test, and the Chi-square tests. Considering the non-normal distribution, urinary perchlorate, nitrate, and thiocyanate were log_2_-transformed. The associations between the urinary levels of perchlorate, nitrate, and thiocyanate and the prevalence of OP were explored using the multivariate logistic regression models. The restricted cubic splines were used to describe the non-linear relationship. If the non-linear relation existed, a piecewise regression analysis based on the logistic regression models was performed to determine the inflection point. Subgroup analyses were performed to investigate whether the association was modified by gender, smoking habits, and races in the fully adjusted model. The R version 3.6 was used for statistical analysis with a *P* < 0.05 set as statistically significant.

## Results

The present study included 13,554 participants (mean age: 48.3 years old, male/female: 6,664/6,890). The median value for urinary perchlorate, nitrate, and thiocyanate were 3.43 (1.89 [first quartile]−6.02 [third quartile]) ng/ml, 45.3 (26.3–70.8) mg/l, and 1.12 (0.52–2.55) mg/l, respectively. There were 3,129 OP cases. In patients with OP, the levels of perchlorate, nitrate, and thiocyanate were higher (Table 1).

**Table 1:** Characteristics of the study population according to the status of oral pain (OP).

**Variables**	**Non-OP** **(*n* = 10,362)**	**OP** **(*n* = 3,192)**	***P***
Male (%)	5,188 (50.1)	1,476 (46.2)	<0.001
Age, years	49.2 (18.8)	45.6 (17.4)	<0.001
Race (%)			<0.001
Non-Hispanic white	4,868 (47.0)	1,265 (39.6)	
Non-Hispanic black	2,210 (21.3)	841 (26.3)	
Mexican American	1,784 (17.2)	633 (19.8)	
Others	1,500 (14.5)	453 (14.2)	
Education (%)			<0.001
Less than high school	2,615 (26.8)	967 (32.4)	
High school or equivalent	2,252 (23.1)	748 (25.0)	
College or above	4,889 (50.1)	1,273 (42.6)	
PIR (%)			<0.001
<1	1,777 (18.6)	848 (28.4)	
1~3	3,861 (40.3)	1,301 (43.6)	
>3	3,933 (41.1)	834 (28.0)	
BMI, kg/m^2^	28.69 (6.63)	29.07 (7.08)	0.005
Drinking, %	1,343 (50.7)	443 (53.0)	0.273
Smoking, %			<0.001
Never	5,323 (74.3)	1,438 (61.9)	
Past	334 (4.7)	116 (5.0)	
Current	1,510 (21.1)	770 (33.1)	
Activity, %			0.102
Vigorous	1,019 (17.0)	340 (17.4)	
Moderate	2,544 (42.4)	778 (39.7)	
Inactive	2,438 (40.6)	841 (42.9)	
Hypertension, %	1,953 (19.7)	507 (16.5)	<0.001
Diabetes, %	1,625 (15.7)	492 (15.4)	0.735
CVD, %	1,052 (10.8)	353 (11.8)	0.125
Creatinine, mg/dL	122.5 (77.49)	130.0 (79.8)	<0.001
Perchlorate, ng/mL	3.40 [1.86, 5.98]	3.51 [1.97, 6.17]	0.046
Nitrate, mg/L	44.4 [25.8, 69.6]	47.9 [27.9, 74.2]	<0.001
Thiocyanate, mg/L	1.05 [0.51, 2.33]	1.33 [0.57, 3.58]	<0.001

*Data are presented as n (%), and mean (SD) or median [lower quartile, upper quartile]. PIR, poverty income ratio; BMI, body mass index; CVD, cardiovascular diseases*.

We calculated the OR for each quartile of exposure to study linearity or relationship of exposure with oral pain (Table 2). As shown in Table 2, the urinary perchlorate and nitrate were not significantly associated with OP. However, the urinary thiocyanate was positively associated with the risk of OP across the three models (Model 1: OR = 1.26, 95% CI: 1.21–1.32, *p* = 0.001; Model 2: OR = 1.06, 95% CI: 1–1.12, *p* = 0.046; and Model 3: OR = 1.06, 95% CI: 1–1.13, *p* = 0.049). However, compared with the lowest quartile of urinary thiocyanate, the highest quartile was not associated with the OP in fully adjusted models.

**Table 2:** Association of urinary perchlorate, nitrate, and thiocyanate with the presence of oral pain.

**Subgroup**	**Cases**	***N***	**Model 1**		**Model 2**		**Model 3**	
			**OR [95% CI]**	***P***	**OR [95% CI]**	***P***	**OR [95% CI]**	***P***
**Perchlorate**								
Q1	752	3,402	Ref		Ref		Ref	
Q2	815	3,388	1.11 [0.98, 1.24]	0.090	1.11 [0.99, 1.25]	0.078	1.11 [0.98, 1.25]	0.087
Q3	803	3,383	1.07 [0.95, 1.21]	0.270	1.09 [0.96, 1.23]	0.173	1.09 [0.96, 1.24]	0.188
Q4	822	3,381	1.08 [0.95, 1.23]	0.235	1.08 [0.95, 1.23]	0.203	1.09 [0.95, 1.25]	0.217
Log_2_	3,192	13,554	1.03 [0.98, 1.07]	0.241	1.03 [0.99, 1.08]	0.181	1.03 [0.98, 1.09]	0.189
**Nitrate**								
Q1	737	3,413	Ref		Ref		Ref	
Q2	753	3,366	1.01 [0.89, 1.13]	0.930	0.99 [0.88, 1.12]	0.872	0.96 [0.85, 1.09]	0.524
Q3	817	3,392	1.08 [0.95, 1.23]	0.241	1.03 [0.91, 1.17]	0.640	0.98 [0.86, 1.13]	0.804
Q4	885	3,383	1.18 [1.02, 1.35]	0.025	1.05 [0.91, 1.22]	0.481	0.99 [0.84, 1.16]	0.867
Log_2_	3,192	13,554	1.04 [0.99, 1.08]	0.132	1.00 [0.96, 1.05]	0.879	0.98 [0.93, 1.03]	0.415
**Thiocyanate**								
Q1	726	3,389	Ref		Ref		Ref	
Q2	699	3,427	0.93 [0.82, 1.05]	0.222	0.91 [0.81, 1.02]	0.120	0.90 [0.80, 1.02]	0.105
Q3	743	3,358	1.02 [0.90, 1.15]	0.788	0.93 [0.82, 1.06]	0.266	0.93 [0.81, 1.05]	0.232
Q4	1,024	3,380	1.57 [1.39, 1.78]	0.001	1.03 [0.89, 1.20]	0.657	1.03 [0.88, 1.20]	0.747
Log_2_	3,192	13,554	1.26 [1.21, 1.32]	0.001	1.06 [1.00, 1.12]	0.046	1.06 [1.00, 1.13]	0.049

*Model 1: adjusted for creatinine, age, gender, and race*.

*Model 2: Model 1+ education, PIR, BMI, drinking, smoking, activity, hypertension, diabetes, and cardiovascular diseases*.

*Model 3: Model 2+ mutual adjustment of log_2_-transformed urinary perchlorate, nitrate, and thiocyanate*.

*Perchlorate: Q1 <1.89, 1.89 ≤ Q2 <3.43, 3.43 ≤ Q3 <6.02, and Q4≥6.02*.

*Nitrate: Q1 <26.3, 26.3 ≤ Q2 <45.3, 45.3 ≤ Q3 <70.8, and Q4≥70.8*.

*Thiocyanate: Q1 < .52, .52 ≤ Q2 <1.12, 1.12 ≤ Q3 <2.55, and Q4≥2.55*.

**Figure 2:**
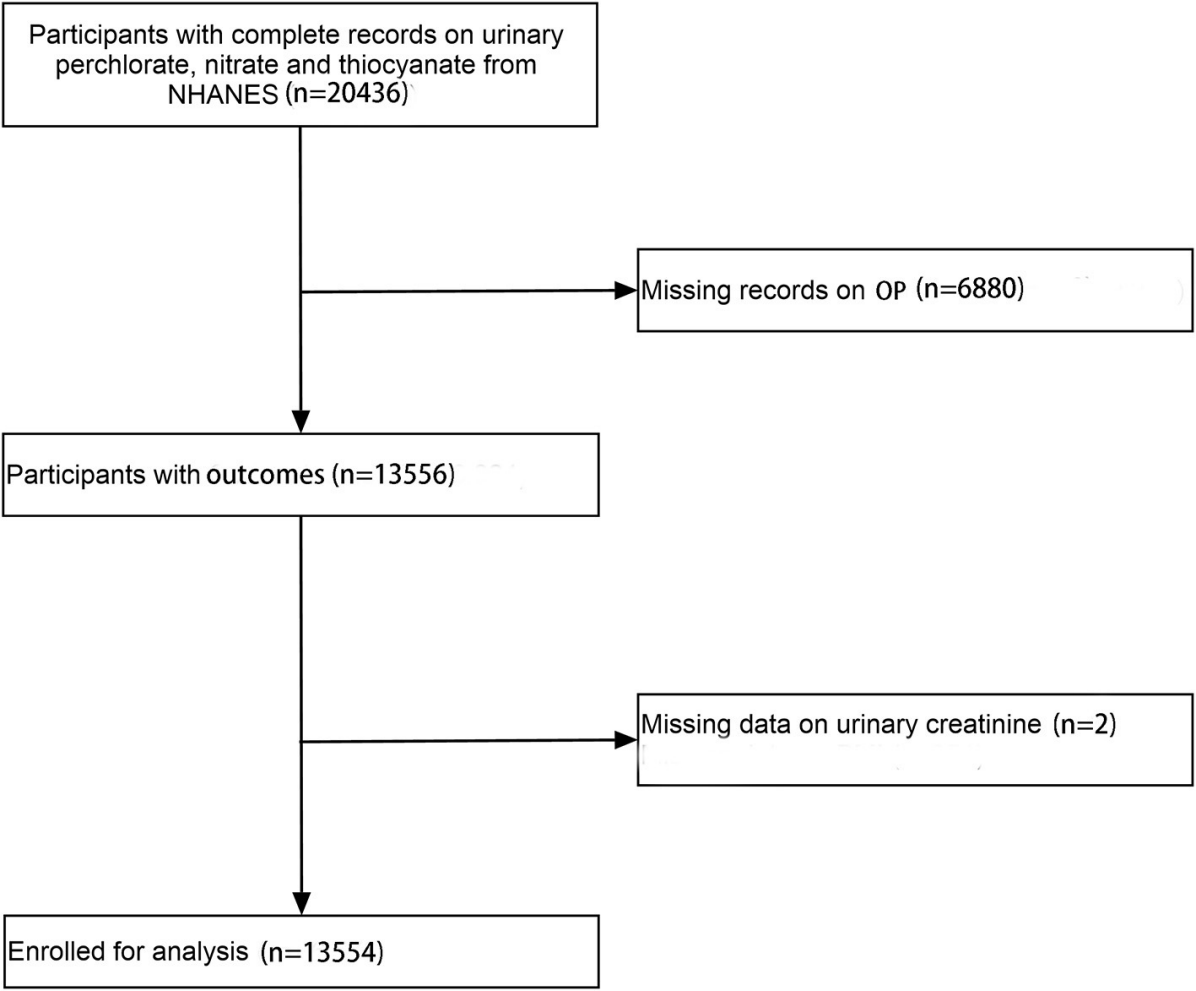
The dose-response analysis between urinary thiocyanate with oral pain. The OR and their 95% CI were represented as association measure. The model was adjusted for creatinine, age, gender, race, education, PIR, BMI, drinking, smoking, activity, hypertension, diabetes, cardiovascular diseases, log_2_-transformed urinary perchlorate, and nitrate. The urinary thiocyanate was log_2_-transformed.

Considering the inconsistency when the urinary thiocyanate was treated as a categorical variable or as a continuous variable, we performed the restricted spline models based on Model 3 (Figure 2), in which the OR was a function of log_2_ (thiocyanate +1). It showed a non-linear association between urinary thiocyanate and OP (*p* for non-linearity = 0.004). Urinary thiocyanate was in a U-shape association with OP, with OR that is higher than 1 for the Log (thicyanate+1).

Considering the non-linearity relation of the urinary thiocyanate with OP, we performed a piecewise regression analysis (Table 3). It showed that the inflection point of the log (thiocyanate +1) was 1 for OP. In addition, the subgroup analysis (Table 4) showed that the association between the urinary thiocyanate with OP was consistent across groups. Even though the association was more evident in men, smokers, and non-Hispanic White, no interaction existed statistically.

**Table 3:** Two-piecewise regression analysis of the effect of thiocyanate on OP.

**Outcomes**	**Inflection point** **[log (thiocyanate+1)]**	**Group**	**OR (95% CI)**
OP	1	≤1.00	0.77 [0.60, 0.99]
		>1.00	1.14 [1.05, 1.22]

**Table 4:** Subgroup analysis of the effect of thiocyanate on OP.

**Subgroup**	**OR**	***P* for interaction**
**Gender**		0.483
Female	1.01 [0.92, 1.11]	
Male	1.10 [1.02, 1.19]	
**Smoking habits**		0.835
No	1.01 [0.92, 1.10]	
Yes	1.16 [1.07, 1.25]	
**Races**		0.135
Non-Hispanic white	1.11 [1.00, 1.22]	
Non-Hispanic black	1.07 [0.96, 1.19]	
Mexican American	0.86 [0.73, 1.02]	
Others	1.02 [0.86, 1.21]	

## Discussion

In this study, we found that the urinary thiocyanate was in a U-shape association with OP. However, the urinary perchlorate and nitrate were not significantly associated with OP. This was the first study to examine the association between perchlorate exposure and OP.

Previous studies have found that the factors associated with OP included non-Hispanic Black, lower-income status (15), depression status (16), and long working hours (17). Our study added a new contribution that the thiocyanate exposure was associated with OP, while the perchlorate or nitrate exposure had no relationship with OP. Thiocyanate level was higher in the saliva of smokers with chronic periodontitis (18). lvY investigated the relationships of different urinary chemical concentrations and adult oral health and found that urinary thiocyanate was related to teeth bone loss and loose (19). Compared with it, our study included a relatively large sample and further explored the non-linear relationship. Interestingly, we found a U-shaped association between urinary thiocyanate and oral pain, namely, a lower and a higher level of urinary thiocyanate; this association increased the risk of oral pain.

The underlying mechanisms deserved further investigation. Firstly, thiocyanate is a metabolite of cyanide in tobacco and is detectable in saliva (20, 21) and in the oral mucosa (6), which could influence the oral microenvironment. Secondly, the thiocyanate may have an antibacterial and antioxidative role for cardiovascular diseases and respiratory viral infections (22, 23), but may aggravate the inflammation responses in the autoimmune disease and gastrointestinal disease (24, 25). Therefore, its effect on oral health could be related to the level of thiocyanate or the comorbidities.

However, several limitations existed in our study. Firstly, the single measurement of urinary thiocyanate might not be representative of long-term exposure. Secondly, this was a cross-sectional study. Thirdly, the OP was self-reported. Finally, diet exposure could influence oral health.

In summary, the thiocyanate exposure was in a U-shape association with the risk of OP. Further regulation of environmental chemicals might need to be considered in the prevention of adult oral health.

## Data Availability Statement

The datasets presented in this study can be found in online repositories. The names of the repository/repositories and accession number(s) can be found at: NHANES.

## Ethics Statement

The studies involving human participants were reviewed and approved by NCHS. The patients/participants provided their written informed consent to participate in this study.

## Author Contributions

JY designed the study. JG performed statistical analysis. HZ and XC wrote the manuscript. All authors contributed to the article and approved the submitted version.

## Conflict of Interest

The authors declare that the research was conducted in the absence of any commercial or financial relationships that could be construed as a potential conflict of interest.

## Publisher's Note

All claims expressed in this article are solely those of the authors and do not necessarily represent those of their affiliated organizations, or those of the publisher, the editors and the reviewers. Any product that may be evaluated in this article, or claim that may be made by its manufacturer, is not guaranteed or endorsed by the publisher.
